# Maternal infections and medications in pregnancy: how does self-report compare to medical records in childhood cancer case–control studies?

**DOI:** 10.1093/ije/dyad019

**Published:** 2023-02-27

**Authors:** Audrey Bonaventure, Eleanor Kane, Jill Simpson, Eve Roman

**Affiliations:** Université Paris Cité and Université Sorbonne Paris Nord, Inserm, INRAE, Center for Research in Epidemiology and StatisticS (CRESS), Paris, France; Epidemiology and Cancer Statistics Group, Department of Health Sciences, University of York, York, UK; Epidemiology and Cancer Statistics Group, Department of Health Sciences, University of York, York, UK; Epidemiology and Cancer Statistics Group, Department of Health Sciences, University of York, York, UK; Epidemiology and Cancer Statistics Group, Department of Health Sciences, University of York, York, UK

**Keywords:** Case–control studies, epidemiologic methods, recall, reliability and validity, prenatal exposure, maternal health, cancer, maternal infection, medication

## Abstract

**Background:**

Studies examining the potential impact of mothers’ health during pregnancy on the health of their offspring often rely on self-reported information gathered several years later. To assess the validity of this approach, we analysed data from a national case–control study of childhood cancer (diagnosed <15 years) that collected health information from both interviews and medical records.

**Methods:**

Mothers’ interview reports of infections and medications in pregnancy were compared with primary care records. Taking clinical diagnoses and prescriptions as the reference, sensitivity and specificity of maternal recall along with kappa coefficients of agreement were calculated. Differences in the odd ratios estimated using logistic regression for each information source were assessed using the proportional change in the odds ratio (OR).

**Results:**

Mothers of 1624 cases and 2524 controls were interviewed ∼6 years (range 0–18 years) after their child’s birth. Most drugs and infections were underreported; in general practitioner records, antibiotic prescriptions were nearly three times higher and infections >40% higher. Decreasing with increasing time since pregnancy, sensitivity was ⩽40% for most infections and all drugs except ‘anti-epileptics and barbiturates’ (sensitivity 80% among controls). ORs associated with individual drug/disease categories that were based on self-reported data varied from 26% lower to 26% higher than those based on medical records; reporting differences between mothers of cases and controls were not systematically in the same direction.

**Conclusions:**

The findings highlight the scale of under-reporting and poor validity of questionnaire-based studies conducted several years after pregnancy. Future research using prospectively collected data should be encouraged to minimize measurement errors.

Key Messages

- Maternal interview reports of infection and medication in pregnancy of 1624 children (0–14 years) with cancer and 2524 age- and sex-matched controls is compared with information recorded in primary care records.
- Infections and medications were substantially underreported at interview, with more than two out of three mothers not reporting a drug that was prescribed in pregnancy.
- Children were, on average, 6.3 years old (range 0–18 years) when their mother was interviewed and the sensitivity of the interview reports was ⩽40% for most infections and all drugs except ‘anti-epileptics and barbiturates’, decreasing with increasing time since pregnancy.
- Odds ratios (ORs) based on questionnaire data varied by –26% to +26% compared with ORs derived from medical record data.
- Reporting differences were detected between mothers of cases and controls, but were not systematically in the same direction.

## Introduction

Speculation that *in utero* exposure to maternal illnesses and/or medications may increase the risk of cancer in children and young adults has been a recurring topic of public concern and scientific debate for >50 years.^1–4^ The rarity of cancer at young ages means that much of the available data come from case–control studies within which mothers are questioned about historical exposures, which often occurred several years in the past. However, despite concerns about the quality of recalled information on maternal illness and medications in pregnancy, relatively few studies have assessed its validity and reliability in the context of childhood cancers.^5–7^ Indeed, even though a number of *in utero* exposures have been reported to be associated with increased risk of cancer in children and young adults,^8–10^ much of the available information about the validity and reliability of recalled obstetric events comes from studies examining other adverse health topics. Generally validated by comparison with obstetric and/or hospital/insurance claims prescription records, self-reported mode of delivery (e.g. caesarean section), gestational age categories (e.g. pre-term) and birthweight are among the most accurately reported,^5^^,^^7^^,^^11–14^ whereas minor illnesses and treatments for episodes of acute illness tend to be poorly recalled.^15–20^ However, validation of mothers’ recall is generally lacking for the illnesses and medications routinely managed in primary care settings that are of interest in studies of childhood cancer.

With a view to providing more specific information to assess the impact of recall errors on the associations observed in studies of childhood cancer, the present report is based on data from the United Kingdom Childhood Cancer Study (UKCCS)—a case–control study that collected information from multiple sources, including maternal interviews and primary care records.

## Methods

Data are from the UKCCS—a national case–control study conducted in the early 1990s across England, Scotland and Wales. Study design and participants’ selection have been detailed previously^9^^,^^21^^,^^22^ and all publications, questionnaires and data abstraction forms are on the study website (www.UKCCS.org). Briefly, the mothers of 3832 children diagnosed with cancer were interviewed (87% of total diagnoses), as were the mothers of 7615 control children who were randomly selected from primary care registers and matched to cases on sex, year and month of birth, and UKCCS region of residence. Face-to-face interviews conducted by trained interviewers with the mothers included a series of closed questions about medications taken in the 3 months before or during pregnancy, with additional questions prompting recall of drug names, times and indications. Drug categories were antibiotics or antibacterial drugs, with separate items asking about penicillin, chloramphenicol, erythromycin, co-trimoxazole or other sulphonamides, and other antibiotics; tranquilizers, antidepressants or sleeping or nerve pills: diazepam, nitrazepam or other; anti-sickness pills; anti-epileptics; phenobarbitone or other barbiturates; hormone, steroid tablets or injections, excluding the contraceptive pill; and any vaccination received during pregnancy.

For maternal infections, questions referred specifically to the pregnancy period: initial questions about rubella (German measles), measles, varicella (chickenpox), shingles, mumps, glandular fever, pneumonia, influenza, cystitis or kidney infections were followed by a general question on any other infections. Additionally, an open-ended question enquired about any other illness or condition requiring a medical visit during the index pregnancy. Data were entered and coded to the International Statistical Classification of Diseases and Related Health Problems, Tenth Revision (ICD-10) for symptoms and illnesses, and to the British National Formulary (BNF Number 24, September 1992 edition) for medications. Area-based deprivation (IMD—index of multiple deprivation, income domain) was derived from the address of residence at birth linked to data from the national 1991 census, using standard methods.^21^

At interview, parents were asked for consent to access their medical notes and 7 of the 10 UKCCS study regions subsequently abstracted information from general practitioner (GP) records. However, the methods adopted within these regions varied—several only abstracted one control per case and some only targeted leukaemias; full details of the methods employed are published elsewhere.^22^^,^^23^ Overall, the primary care records of the mothers of 1718 cases and 2633 controls were located and abstracted, and after excluding incomplete records, 1624 cases (94.5%) and 2524 controls (95.9%) were included in the present analyses.^9^

Primary care data were abstracted from written medical records using standardized operating procedures and forms; symptoms and illnesses were coded to ICD-10 and drugs to the BNF. To enable data sources to be compared, medications and infections listed in primary care records were subsequently grouped into the questionnaire categories, with the definition for ‘any infection’ being restricted to unequivocal diagnoses of certain infections. Supplementary Table S1 (available as Supplementary data at *IJE* online) shows the classification of drugs and infections used for this analysis.

### Statistical analyses

In order to assess the relative change in the odds ratios (ORs) of childhood cancer, unconditional logistic regressions were conducted separately on interview and GP data, adjusting for factors a priori suspected to affect the quality of recall: child’s age at interview, pregnancy order, maternal age at birth and deprivation quintile of residence, as a proxy for the household’s socio-economic status at the time of the child’s birth. We derived the absolute change in the OR as the difference between the adjusted OR for interview and the adjusted OR for GP data; the relative change in the OR was calculated as the difference in ORs divided by the adjusted GP OR. To examine whether the information reported by mothers was similar to that recorded in GP records, sensitivity and specificity were calculated for interview data, using medical records as the reference; separate calculations were made for mothers of cases and those of controls. We also calculated unweighted kappa statistics with their 95% CIs. Analyses were performed using the SAS 9.4 (SAS Institute, Cary, North Carolina, USA) software.

## Results

The characteristics of cases and controls included in this study are distributed in Table 1. Whereas age at diagnosis/pseudodiagnosis was similarly distributed between cases and controls, cases tended to be slightly younger at the time of their mother’s interview (cases’ mean age 5.8 years, controls’ mean age 6.6 years), reflecting the efforts made to include case children as promptly as possible following their diagnosis. At the time of the index birth, mothers of cases tended to live in less affluent areas (χ^2^*P *=* *0.02) and they were, on average, younger than control mothers (χ^2^*P *=* *0.02), but no case–control differences with pregnancy order were evident.

**Table 1:** Characteristics of cases and controls with primary care abstractions

	Controls		Cases	
	*n*	%	*n*	%
**Total**	2524		1624	
**Age of child at diagnosis/pseudodiagnosis (years)**				
≤4	1357	53.8%	878	54.1%
5–9	653	25.9%	421	25.9%
10–14	514	20.4%	325	20.0%
Mean (CI)	5.3 (5.1–5.4)		5.2 (5.0–5.4)	
**Age of child at interview (years)**				
≤4	1015	40.2%	780	48.0%
5–9	847	33.6%	473	29.1%
10–14	566	22.4%	348	21.4%
≥15	96	3.8%	23	1.4%
Mean (CI)	6.6 (6.4–6.7)		5.8 (5.6–6.0)	
**Pregnancy order**				
1	841	33.3%	549	33.8%
2	850	33.7%	521	32.1%
3	471	18.7%	291	17.9%
4+	362	14.3%	263	16.2%
**Maternal age at birth (years)**				
<25	721	28.6%	498	30.7%
25–29	904	35.8%	616	37.9%
>29	899	35.6%	510	31.4%
Mean (CI)	27.7 (27.5–27.9)		27.2 (27.0–27.5)	
**Area-based deprivation at birth**				
Affluent 1	529	21.0%	314	19.3%
2	553	21.9%	323	19.9%
3	561	22.2%	334	20.6%
4	480	19.0%	342	21.1%
Deprived 5	393	15.6%	303	18.7%
Missing	8	0.3%	8	0.5%

Generally, far fewer medications and infections were reported at interview than were recorded in primary care records (Table 2). Specifically, antibiotic prescriptions were almost three times higher in GP records (control mothers 33.7%; case mothers 33.8%) than in interview data (control mothers 12.3%; case mothers 12.5%). Likewise, whereas ∼38% of case and control mothers were diagnosed with an infection, at interview only 28% reported having had one. However, in contrast to prescription drugs, which were universally higher in GP records than in interview reports, infections of the urinary tract (cystitis or kidney) and influenza were both reported more frequently at interview: urinary tract infections (controls: 10.4% interview, 5.0% GP, χ^2^*P *<* *0.0001; cases: 10.2% interview, 4.4% GP, χ^2^*P *<* *0.0001) and influenza (controls: 5.7% interview, 1.6% GP, Fisher’s exact test *P *=* *0.007; cases: 5.8% interview; 1.5% GP, Fisher’s exact test *P *=* *0.002).

**Table 2:** Odds ratios (ORs) for childhood cancer and *in utero* exposure to prescription drugs and infections: general practitioner (GP) records vs interview questionnaires

	Questionnaire data				GP records				Change in OR^a^	
	Controls 2524	Cases 1624	OR (95% CI)	Adj OR (95% CI)	Controls 2524	Cases 1624	OR (95% CI)	Adj OR (95% CI)	Absolute	Relative
	*N* (%)	*N* (%)			*N* (%)	*N* (%)				
**Drugs in the 3 months before or during pregnancy**										
Antibiotic, antibacterial	301 (12.3)	195 (12.5)	1.02 (0.84–1.23)	0.96 (0.79–1.17)	850 (33.7)	549 (33.8)	1.01 (0.88–1.15)	0.95 (0.83–1.09)	0.01	1%
Penicillin	105 (4.4)	66 (4.4)	0.99 (0.73–1.36)	0.92 (0.67–1.26)	610 (24.2)	412 (25.4)	1.07 (0.92–1.23)	1.02 (0.88–1.18)	–0.10	–10%
Other	258 (10.6)	176 (11.3)	1.07 (0.88–1.32)	1.03 (0.84–1.26)	369 (14.6)	247 (15.2)	1.05 (0.88–1.25)	1.00 (0.84–1.20)	0.03	3%
Anti-sickness	99 (3.9)	72 (4.5)	1.14 (0.83–1.55)	1.18 (0.87–1.62)	143 (5.7)	102 (6.3)	1.12 (0.86–1.45)	1.13 (0.87–1.48)	0.05	4%
Hormones, steroids^b^	70 (2.8)	30 (1.9)	0.66 (0.43–1.02)	0.66 (0.43–1.02)	101 (4.0)	57 (3.5)	0.87 (0.63–1.21)	0.89 (0.64–1.24)	–0.23	–26%
Tranquilizers, antidepressants^c^	54 (2.1)	36 (2.2)	1.04 (0.68–1.59)	1.03 (0.66–1.58)	79 (3.1)	68 (4.2)	1.35 (0.97–1.88)	1.37 (0.98–1.91)	–0.34	–25%
Vaccines	35 (1.4)	25 (1.6)	1.13 (0.68–1.90)	1.12 (0.66–1.88)	56 (2.2)	39 (2.4)	1.09 (0.72–1.64)	1.02 (0.67–1.55)	0.10	10%
Anti-epileptics, barbiturates	20 (0.8)	13 (0.8)	1.01 (0.50–2.04)	1.04 (0.51–2.11)	15 (0.6)	9 (0.6)	0.93 (0.41–2.14)	0.99 (0.43–2.28)	0.05	5%
**Infections during pregnancy**										
Any infection	689 (27.3)	473 (29.1)	1.09 (0.95–1.26)	1.05 (0.91–1.20)	968 (38.7)	597 (37.3)	0.94 (0.83–1.07)	0.91 (0.79–1.03)	0.14	15%
Cystitis, kidney	260 (10.4)	165 (10.2)	0.99 (0.80–1.21)	0.89 (0.72–1.10)	126 (5.0)	70 (4.4)	0.86 (0.64–1.16)	0.84 (0.62–1.14)	0.05	6%
Influenza	143 (5.7)	94 (5.8)	1.02 (0.78–1.34)	0.98 (0.75–1.29)	41 (1.6)	24 (1.5)	0.92 (0.55–1.52)	0.85 (0.50–1.43)	0.13	15%
Rubella, measles, varicella, shingles, mumps, glandular fever^d^	27 (1.1)	14 (0.9)	0.80 (0.42–1.54)	0.79 (0.41–1.51)	15 (0.6)	10 (0.6)	1.04 (0.47–2.33)	0.99 (0.44–2.24)	–0.20	–20%
Infection, other^e^	350 (13.9)	254 (15.6)	1.15 (0.97–1.37)	1.15 (0.96–1.37)	879 (35.1)	542 (33.9)	0.95 (0.83–1.08)	0.91 (0.80–1.04)	0.24	26%

Participants with missing data excluded from analyses. Adjustment on pregnancy order, child's age at interview, maternal age at birth and deprivation quintile.

a Change in adjusted ORs calculated using the GP medical record as reference. Absolute change: ORq-ORm; relative change: ORq-ORmORm.

b Hormone, steroid tablets or injections, excluding contraceptives.

c Also includes sleep or nerve pills.

d Specific infections asked about at interview.

e Infection, other: any infection other than influenza, cystitis, kidney infection, rubella, measles, varicella, shingles, mumps or glandular fever (see Supplementary Table 1, available as Supplementary data at *IJE* online).

ORs estimated from interview data varied by –26% to +26% (–0.34 to +0.24 in absolute difference) when compared with those generated from GP data (Table 2). For specific medications, the change in the adjusted OR was largest for hormonal treatments (–26%) and tranquilizers (–25%); for infections, the change was most marked for the group of infections that were specifically asked about at interview (–20%) but no consistent pattern was evident. For all but five exposures (antibiotics, tranquilizers/antidepressants, any infection, cystitis/kidney infection, influenza), the OR estimated from the questionnaire data was further away from unity than the OR estimated from the GP record. The OR for ‘any infection’ was <1 using GP record data but >1 using interview data, although the overlapping CIs both included 1.

More importantly, perhaps, comparing individual reports (Table 3) revealed that the number of mothers who failed to report a drug prescription (false negative, –/+) was greater than the number who accurately reported a drug that had been prescribed (true positive, +/+); this led to self-report for the drugs of interest generally having a low sensitivity. Specificity on the other hand was >90% for all drug categories. Anti-epileptics and barbiturates were the only accurately reported drug category, with a sensitivity of 80.0% in the control group and 88.9% in the case group; conversely, for antibiotics, the sensitivity of self-report was ∼23% and that for penicillin was noticeably lower (9%). The agreement between maternal report and prescription record was substantial for anti-epileptics and barbiturates [kappa of 0.68 (95% CI: 0.51–0.86) in the control group and 0.73 (95% CI: 0.51–0.94) in the case group] but fair to poor for the other drug categories: kappa ranging from 0.43 (95% CI: 0.33–0.52) for hormonal treatments down to 0.09 (95% CI: 0.06–0.13) for penicillin in the controls [0.40 (95% CI: 0.27–0.54) and 0.09 (95% CI: 0.05–0.14), respectively, for cases]. The measures of validity and agreement for drugs were broadly similar for cases and controls.

**Table 3:** Distribution of medications and infections in general practitioner (GP) records and reported at interview; the sensitivity, specificity, kappa and 95% CI of self-report are calculated relative to the GP records

	Controls (*n* = 2524)							Cases (*n* = 1624)						
	Questionnaire/GP records							Questionnaire/GP records						
	Agreement		Disagreement		Sens^a^	Spec^a^	Kappa	Agreement		Disagreement		Sens^a^	Spec^a^	Kappa
	+/+	–/–	–/+	+/–	(%)	(%)	(95% CI)	+/+	–/–	–/+	+/–	(%)	(%)	(95% CI)
**Drugs in 3 months before or during pregnancy**														
Antibiotic, antibacterial	7.6%	61.8%	25.9%	4.7%	22.7%	92.9%	0.19 (0.15–0.22)	7.7%	61.7%	25.8%	4.7%	23.0%	92.9%	0.19 (0.14–0.23)
Penicillin	2.3%	74.1%	21.5%	2.1%	9.5%	97.2%	0.09 (0.06–0.13)	2.4%	72.7%	22.9%	2.0%	9.4%	97.3%	0.09 (0.05–0.14)
Other	2.7%	77.6%	11.8%	7.9%	18.4%	90.8%	0.10 (0.06–0.15)	3.6%	77.7%	11.1%	7.6%	24.8%	91.1%	0.18 (0.11–0.24)
Anti-sickness	1.8%	92.1%	3.9%	2.2%	31.0%	97.7%	0.33 (0.25–0.41)	1.6%	90.9%	4.6%	2.9%	26.0%	97.0%	0.26 (0.17–0.36)
Hormones, steroids^b^	1.5%	94.7%	2.5%	1.3%	38.0%	98.7%	0.43 (0.33–0.52)	1.1%	95.8%	2.4%	0.7%	32.1%	99.2%	0.40 (0.27–0.54)
Tranquilizers, antidepressants^c^	0.9%	95.6%	2.3%	1.3%	27.8%	98.7%	0.31 (0.21–0.42)	0.6%	94.2%	3.6%	1.6%	14.7%	98.3%	0.17 (0.07–0.27)
Vaccines	0.4%	96.7%	1.9%	1.0%	16.1%	98.9%	0.18 (0.07–0.29)	0.4%	96.6%	1.8%	1.1%	20.0%	98.8%	0.22 (0.08–0.36)
Anti-epileptics, barbiturates	0.5%	99.1%	0.1%	0.3%	80.0%	99.7%	0.68 (0.51–0.86)	0.5%	99.1%	0.1%	0.3%	88.9%	99.7%	0.73 (0.51–0.94)
**Infections during pregnancy** ^d^														
Any infection	14.7%	48.7%	24.0%	12.7%	37.9%	79.3%	0.18 (0.14–0.22)	15.0%	48.5%	22.3%	14.2%	40.2%	77.4%	0.18 (0.13–0.23)
Cystitis, kidney	1.8%	86.3%	3.3%	8.6%	35.7%	90.9%	0.18 (0.12–0.24)	1.8%	87.2%	2.6%	8.4%	40.6%	91.2%	0.19 (0.12–0.27)
Influenza	0.3%	93.0%	1.3%	5.4%	17.5%	94.5%	0.05 (0.00–0.11)	0.4%	93.0%	1.1%	5.5%	25.0%	94.5%	0.08 (0.01–0.16)
Rubella, measles, varicella, shingles, mumps, glandular fever^e^	0.5%	98.8%	0.1%	0.6%	80.0%	99.4%	0.57 (0.39–0.75)	0.4%	99.0%	0.2%	0.4%	70.0%	99.6%	0.61 (0.37–0.84)
Infection, other^f^	7.2%	58.3%	27.9%	6.6%	20.5%	89.8%	0.12 (0.08–0.15)	7.5%	57.8%	26.4%	8.3%	22.1%	87.4%	0.11 (0.06–0.16)

a Sens, sensitivity; Spec, specificity (reference GP records).

b Hormone, steroid tablets or injections, excluding contraceptives.

c Also includes sleep or nerve pills.

d 24 cases (1.5%) and 21 controls (0.8%) excluded due to missing information on diagnosis.

e Specific infections asked about at interview.

f Infection, other: any infection other than influenza, cystitis, kidney infection, rubella, measles, varicella, shingles, mumps or glandular fever (see Supplementary Table 1, available as Supplementary data at *IJE* online).

**Figure 1:**
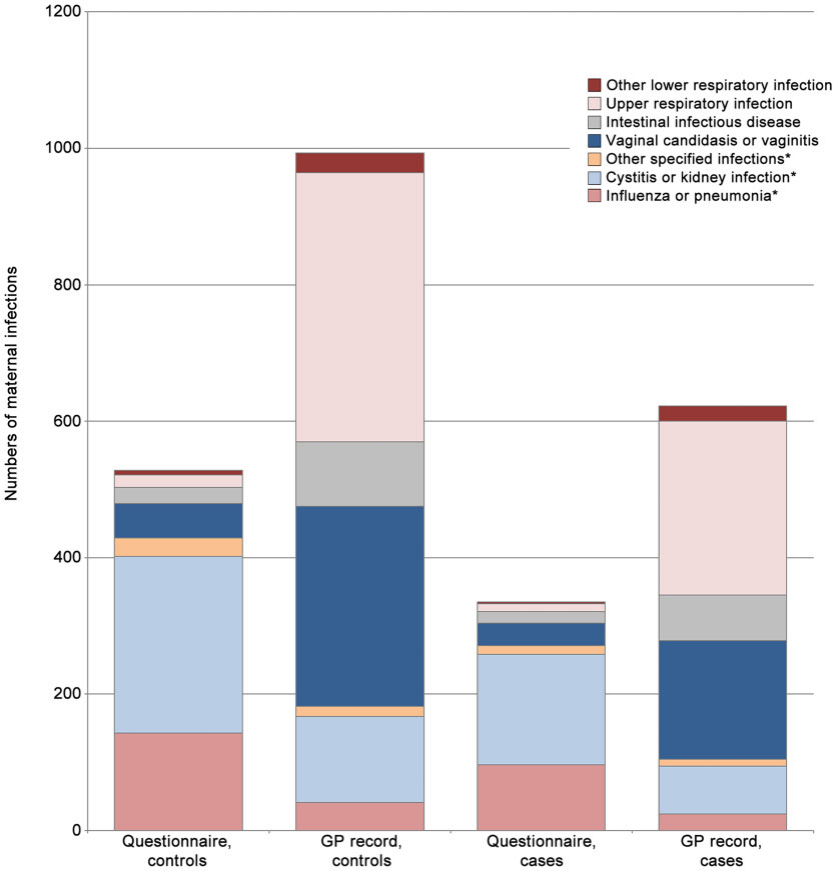
Cumulative numbers of maternal infections in pregnancy by type of illness and data source. *Specific question in the questionnaire. Based on 1600 cases (all cancers combined) and 2503 controls (24 cases and 21 controls excluded due to missing information on diagnosis in medical record). GP, general practitioner

Likewise, more mothers failed to report infections that were recorded in their GP notes (24.0% of controls’ mothers, 22.3% of cases’) than those who accurately recalled having infections (14.7% and 15.0% for mothers of controls and cases, respectively) (Table 3 and Figure 1). The sensitivities of specific infections were low (⩽40%) except for the group of rubella, measles, varicella, shingles, mumps and glandular fever that were specifically asked about during the interview, where sensitivity reached 80.0% in the control group and 70.0% in the case group. Although rare (5 cases and 11 controls), varicella (chickenpox) was well reported, with a sensitivity of 90.9% in controls and 80.0% in cases [kappa 0.66 (95% CI: 0.47–0.86) and 0.67 (95% CI: 0.36–0.97), respectively].

**Figure 2.**
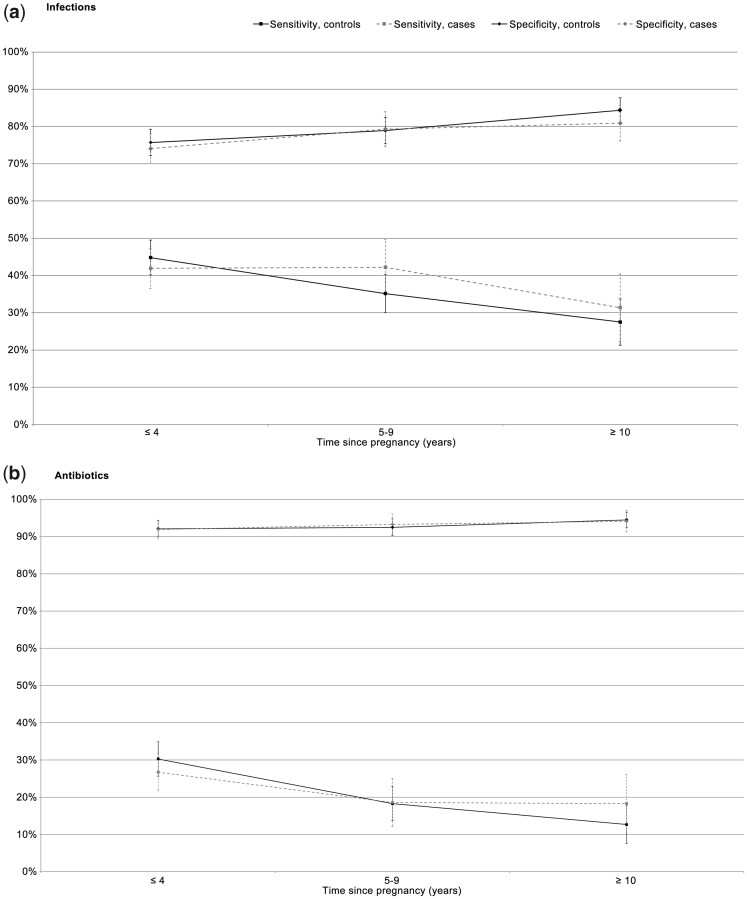
Sensitivity (%) and specificity (%) of self-reported infections (a) and antibiotics use (b) in pregnancy by time since pregnancy (child’s age at interview, in years)

The validity of maternal report worsened as the length of time between the pregnancy and the interview increased; sensitivity for both self-reported infections and antibiotics fell over time, with sensitivity up to 18% lower for interviews performed at least 10 years after the birth compared with those performed in the first 4 years (Figure 2). Conversely, specificity increased by ∼10% for cases and controls for any infection, and it was roughly stable for antibiotics. There were no obvious patterns of trends by pregnancy order, maternal age at birth or deprivation index quintile, as shown in Supplementary Figures S1–S3 (available as Supplementary data at *IJE* online).

## Discussion

In this large childhood cancer case–control study, which included data on >4000 individuals, mothers were interviewed face to face on average ∼6 years after their child’s birth. Comparisons with primary records revealed that mothers’ interview recollections about medications and infections during pregnancy suffered from major under-reporting and low sensitivity, the latter declining rapidly as the interval between pregnancy and interview increased. Sensitivity varied, however, with both drug and infection type: anti-epileptics and barbiturates were more accurately recalled than more commonly prescribed medications, as were the rarer potentially more serious infections like rubella and varicella. Interestingly, although influenza and urinary tract infections were more than twice as likely to be reported at interview than recorded in GP records, self-report was exceptionally poor; 82.5% (33/40) of women who had an influenza diagnosis recorded in their medical records did not report it at interview (sensitivity 17.5%) and likewise 64% (81/126) of those with a confirmed urinary tract infection also failed to report it (sensitivity 35.7%). Interestingly, with sensitivities of >65% for any infection during infancy being reported,^33^ mothers’ recollections of their own infections during pregnancy are poorer than their recall of their child’s infections.

Like other childhood cancer studies that examined recall,^5–7^ we did not observe any evidence of the over-reporting that has long been suspected in mothers of children with serious diseases or conditions.^12^^,^^24^ Consequently, when examining associations between childhood cancer and maternal infections and drugs during pregnancy, our risk estimates based on self-report were not inflated by this source of differential bias. We did however find that recall errors impacted on our case–control comparisons of maternal infections and medications, with risk estimates based on the two data sources differing by up to 26%. Where there is non-differential misclassification of a dichotomous exposure, it is generally accepted that the risk estimates will be biased towards the null. Interestingly, however, risk estimates based on self-report were mostly further away from unity than those based on GP records, suggesting some differential misclassification. No obvious systematic pattern in accuracy of the recall between case and control mothers was, however, detected, with sensitivity higher in cases for some drugs and infections, and higher in controls for others. Nonetheless, it is worth noting that larger studies and pooled/meta-analyses could produce sufficient numbers and statistical power to detect small effects for all but the rarest of exposures, potentially leading to incorrect conclusions.

Most previous investigations of mothers’ recall have compared maternal reports to obstetric,^5^^,^^7^^,^^12^ hospital^6^^,^^19^ or hospital/insurance claims prescription records.^15–17^^,^^20^^,^^25^ By using primary care records, we have evaluated the validity and reliability of mothers’ recall for infections and medications that have not previously received such attention, despite the aetiological interest in relation to childhood cancers^8^^,^^10^^,^^26^ as well as other adverse health outcomes.^27–32^ Considering the general nature of the events examined, we observed some similarities with others: e.g. in agreement with our findings for anti-epileptics and barbiturates, drugs that are routinely taken for chronic conditions have been found to be more accurately reported than those taken for acute illnesses, whereas, in line with our low specificity for drugs such as penicillin, acute diseases, conditions with complicated names or drugs taken for a short period of time tend to be poorly recalled.^14–17^^,^^20^^,^^25^ Others too have observed that mothers’ recall worsens as the birth in question becomes more distant in time^5^ but even in studies in which mothers were interviewed during pregnancy or shortly after birth, most illnesses and medications show poor agreement with medical records.^15–17^^,^^19^^,^^20^

A major strength of this study is the ability to compare data collected at interview with information abstracted directly from medical records; although our study was conducted in the early 1990s, it remains unique as there is no UK-wide linkage of children’s birth records to their mother’s primary care data. In the context of the UK’s universal healthcare system in which antenatal care is managed in primary care, health events are recorded at the time in question and so findings are not subject to the reporting and recall biases commonly associated with self-reported information about past events. As such, we were able to examine conditions that required a primary care visit along with any medications prescribed by the GP, which would not be subject to prescription charges during the pregnancy. Nonetheless, as not all illnesses and conditions necessitate a GP visit, minor infections, such as common colds, will be missed. On the other hand, our study only examined infections and medications in GP notes, and as such was not designed to examine severe maternal infections. With respect to medications, recall errors seem the most likely explanation for the high proportion of mothers who did not report a prescribed medication rather than other reasons such as issues of compliance.^34^^,^^35^ Furthermore, we found little evidence for differential selection of case and control families based on participation or agreement for medical record abstraction, which could have affected our findings. Our measures of absolute and relative (proportional) changes in the OR might not have highlighted the influence of recall error due to a lack of association between exposures and disease; where stronger associations are observed, absolute and relative changes in the OR should be able to highlight the full impact of recall bias.

## Conclusion

This work highlights the important limitations of maternal self-report as a source of information about maternal infections and prescription drugs in pregnancy, in the context of childhood cancer case–control studies. Future work should avoid, as much as possible, relying solely on questionnaire data for information about women’s health in pregnancy, particularly for pregnancies that occurred several years in the past. Whereas gathering medical record data poses undeniable challenges, the use of data sources not subject to report bias, such as medical records, birth certificates and other medico-administrative data collected prospectively, should be encouraged by medical and scientific bodies, and supported by funders and political authorities as a way to improve the quality of the research.

## Ethics approval

This study involves human participants and was approved by Yorkshire & the Humber-Leeds West Research Ethics Committee (reference 18/YH/0135).

## Supplementary Material

dyad019_Supplementary_Data

## Data Availability

No data are available. Although ethical permissions and agreements with providers of national data mean that data deemed to have the potential to identify individuals cannot be transferred or accessed off-site, UKCCS data are contributing to several ongoing research projects. For information on how to collaborate with UKCCS researchers and investigate questions of interest, please e-mail the Principal Investigator (E.R.).
