# Supplementary material for: Maternal infections and medications in pregnancy: how does self-report compare to medical records in childhood cancer case–control studies?
Source: Int J Epidemiol. 2023 Feb 27;52(4):1187–96. doi: 10.1093/ije/dyad019 (PMC10396422; doi:10.1093/ije/dyad019)

**Table S1**: Classification of drugs and infections used for this analysis

| Drug category | BNF codes |
|---|---|
| Any antibiotic or antibacterial | 5.1, 11.3.1, 13.10.1, 7.2.2/09, 12.1.1/04, 12.1.1/05, 12.1.1/07, 12.1.1/08, 12.1.1/09, 12.1.1/10, 13.6/02 |
| Penicillins | 5.1.1 |
| Anti-sickness pills | 4.6 |
| Hormone, steroid tablets or injections (excl. contraceptive pill) | 6.x, 8.3, 7.1.1, 10.1.2, 9.1.3/00 |
| Tranquilizers, antidepressants, sleep or nerve pills: hypnotics, anxiolytics, antipsychotics, antidepressants | 4.1.1, 4.1.2, 4.2, 4.3 |
| Anti-epileptics, phenobarbitone or other barbiturates | 4.8, 4.1.3, 15.1.1 |
| Vaccines or immunoglobulins | 14.4, 14.5 |

| Disease category | ICD-10 codes |
|---|---|
| Any infection | A00-B99, E06.0, E32.1, G00-G08 , H00.0, H05.0, H60, H66, I88, I89.1, J00-J06, J09-J18, J20-J22, J32, J34.0, J35.0, J36, J40, K02.9, K04.6, K04.7, K05.6, K11.2, K11.3, K12.0, K12.2, K37, K81, L00-L03, L05, L08, N10-N12, N30.9, N39.0, N61, N70, N72, N73.9, N75-N76, O23, O86, O91, T81.4. |
| Cystitis, kidney infection | N10.X, N12.X, N30.9, N39.0, O23.0, O23.1, O23.2, O23.4, O23.9 |
| Influenza | J10.0, J11.1 |
| Other specified infections (listed in the questionnaire) | Rubella/German measles (B06.9), measles (B05.9), varicella/chickenpox (B01.9), shingles (B02.9), mumps (B26.9), glandular fever (B27.9, B27.0) |

The codes shown are only those present in the data, and might omit other infection codes absent from the data.

**Figure S1:** Sensitivity (%) and specificity (%) of self-reported infections in pregnancy by pregnancy order

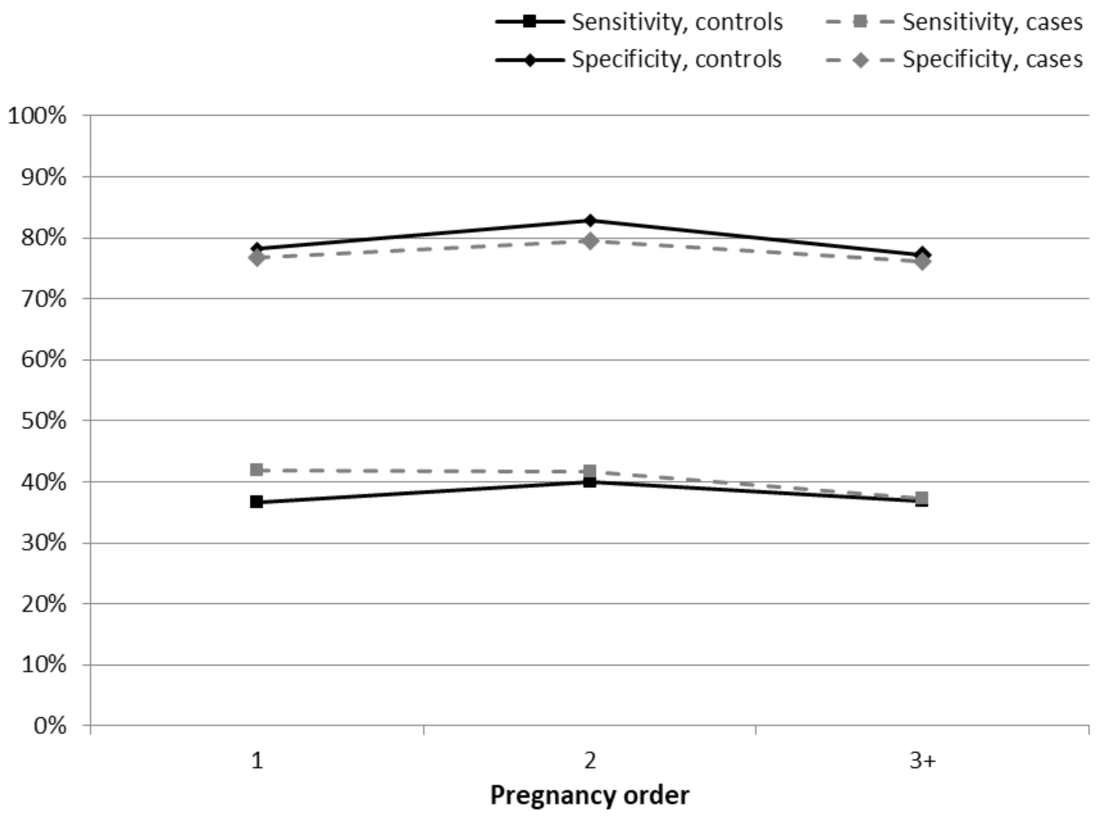

**Figure S2:** Sensitivity (%) and specificity (%) of self-reported infections in pregnancy by maternal age at child's birth (in years)

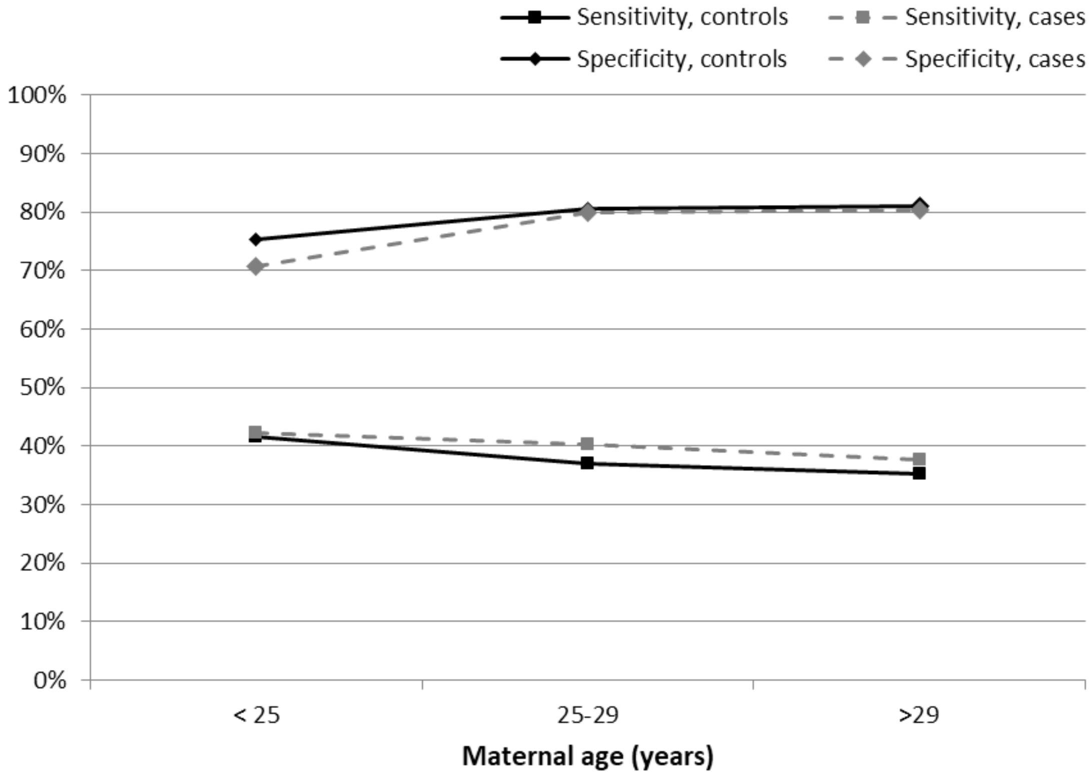

**Figure S3:** Sensitivity (%) and specificity (%) of self-reported infections in pregnancy by quintiles of deprivation index at birth

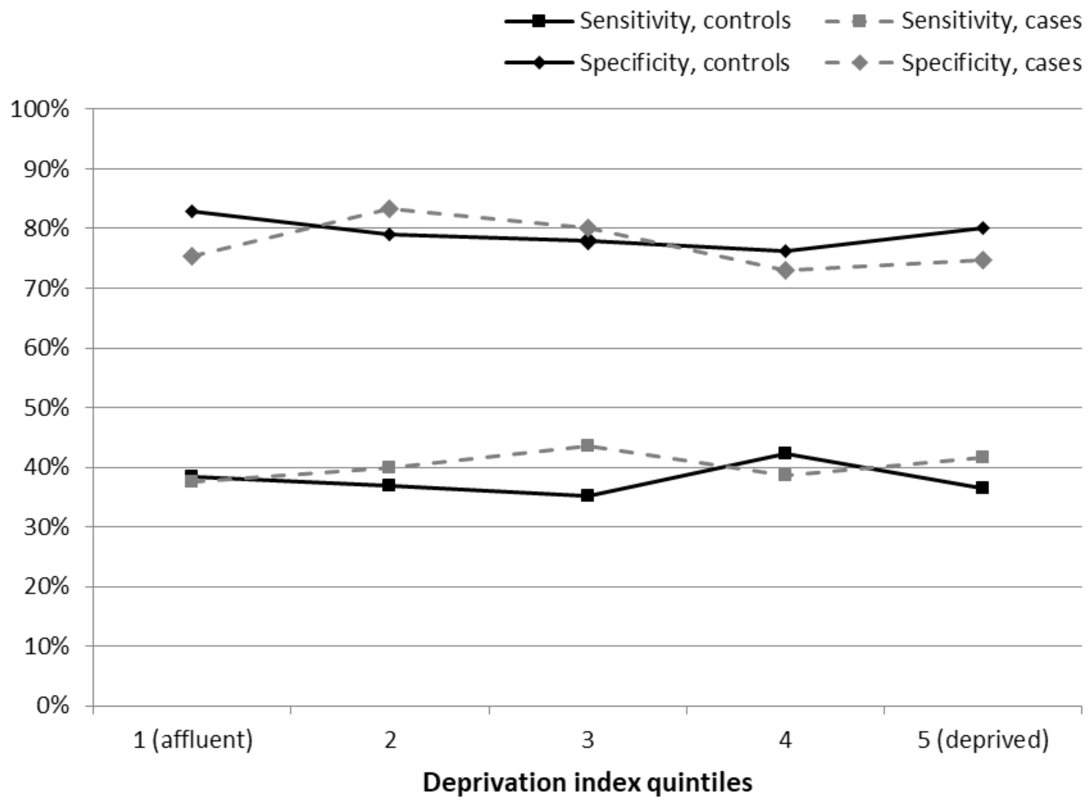

Supplement: dyad019_Supplementary_Data [file dyad019_supplementary_data.pdf]
